# Running Exercise Alleviates Pain and Promotes Cell Proliferation in a Rat Model of Intervertebral Disc Degeneration

**DOI:** 10.3390/ijms16012130

**Published:** 2015-01-19

**Authors:** Shuo Luan, Qing Wan, Haijie Luo, Xiao Li, Songjian Ke, Caina Lin, Yuanyuan Wu, Shaoling Wu, Chao Ma

**Affiliations:** Department of Rehabilitation Medicine, Sun Yat-sen Memorial Hospital, Sun Yat-sen University, Guangzhou 510120, China; E-Mails: luanshuo126@163.com (S.L.); wanqing_2012@163.com (Q.W.); rogerluohj@163.com (H.L.); lixiao204011987@sina.com (X.L.); songjian112626@163.com (S.K.); lincaina826@126.com (C.L.); wyy_98@126.com (Y.W.)

**Keywords:** degenerative disc disease (DDD), running exercise, cell proliferation, disc regeneration

## Abstract

Chronic low back pain accompanied by intervertebral disk degeneration is a common musculoskeletal disorder. Physical exercise, which is clinically recommended by international guidelines, has proven to be effective for degenerative disc disease (DDD) patients. However, the mechanism underlying the analgesic effects of physical exercise on DDD remains largely unclear. The results of the present study showed that mechanical withdrawal thresholds of bilateral hindpaw were significantly decreased beginning on day three after intradiscal complete Freund’s adjuvant (CFA) injection and daily running exercise remarkably reduced allodynia in the CFA exercise group beginning at day 28 compared to the spontaneous recovery group (controls). The hindpaw withdrawal thresholds of the exercise group returned nearly to baseline at the end of experiment, but severe pain persisted in the control group. Histological examinations performed on day 70 revealed that running exercise restored the degenerative discs and increased the cell densities of the annulus fibrosus (AF) and nucleus pulposus (NP). Furthermore, immunofluorescence labeling revealed significantly higher numbers of 5-bromo-2-deoxyuridine (BrdU)-positive cells in the exercise group on days 28, 42, 56 and 70, which indicated more rapid proliferation compared to the control at the corresponding time points. Taken together, these results suggest that running exercise might alleviate the mechanical allodynia induced by intradiscal CFA injection via disc repair and cell proliferation, which provides new evidence for future clinical use.

## 1. Introduction

Chronic low back pain accompanied with intervertebral disc (IVD) degeneration is a prevalent and complicated musculoskeletal disorder [1,2]. Recent epidemiological evidence suggests that up to 70% of the population experiences low back pain at some point in their lives [3], which imposes heavy medical and financial burdens on both patients and society. Despite previous studies that have suggested that various factors, including age, hereditary and environmental factors, might contribute to the degeneration process, the underlying mechanisms of symptomatic degenerative disc disease (DDD) remain poorly understood [4,5]. In recent years, many animal models have offered insight into the complex pathological mechanism of DDD and allowed researchers to conduct efficacy studies of both preventive and therapeutic interventions [6,7]. In 2009, Lee *et al.* [8] proposed a rat model that involved the injection of complete Freund’s adjuvant (CFA) into the 5th/6th lumbar (L5-6) IVD; this model is characterized by degenerative disc changes, mechanical allodynia in the bilateral hindpaws and up-regulation of inflammatory factors. This model successfully mimics clinical situations of chronic low back pain and disc degeneration.

Most international guidelines for low back pain management recommend that an appropriate physical exercise program be implemented to help to relieve pain and recover impaired motor function [9,10]. Previous studies have primarily focused on the therapeutic effects of such programs on the strengthening and stabilization of spine structures from the biomechanical perspective [11]; however, how dynamic mechanical stimulation influences the degenerative discs at the cellular and molecular levels is rarely discussed. Many studies have evaluated the efficacy of physical exercise on cell proliferation in muscles, tendons and nerves, and the results have indicated that cell proliferation and migration are positively influenced by running exercise [12]. For example, treadmill running exercise was proved to induce an anabolic effect on tendons by enhancing tendon stem cells (TSC) proliferation and collagen production [13]. Similarly, stem and progenitor cells have previously been identified and maintained at slow cell turnover rates in the stem cell niche (SN), peripheral epiphyseal cartilage (pEC), outer rings (AFo) and inner rings of the AF (AFi) within the IVDs [14,15]; these findings raise the possibility of promoting cell proliferation and repairing damaged structures with the stimulation of physical exercise. Despite the above findings, the potential analgesic and disc restoration effects of physical exercise on degenerative discs remain unexamined, and furthermore, the relationship between pain relief and cell proliferation also needs verification.

Based on the CFA-induced intervertebral disc degeneration rat model, the aim of the present study was to explore the analgesic effects of an 8-week running protocol via pain-related behavioral measurements, and more importantly, the restorative and therapeutic effects achieved by running exercise. Through comparisons of the recovery processes of the exercise group and spontaneous recovery group, the effects of running exercise on damaged disc repair and cell proliferation in complex degenerative discs were investigated.

## 2. Results and Discussion

### 2.1. Running Exercise Attenuated Intradiscal Complete Freund’s Adjuvant (CFA)-Induced Mechanical Allodynia

**Figure 1:**
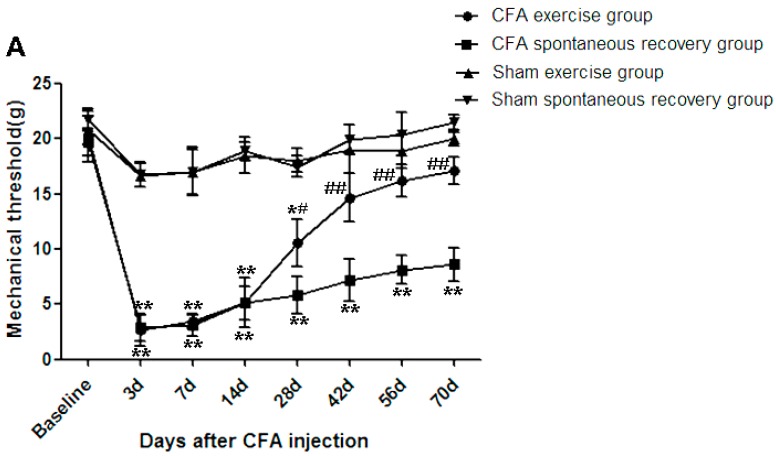

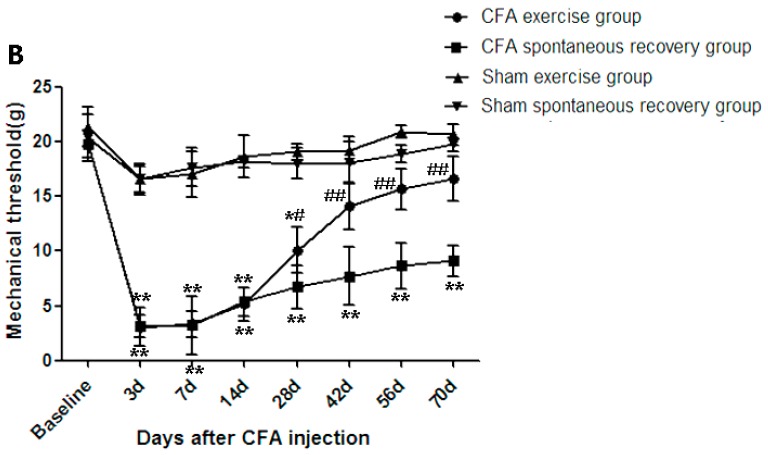
Running exercise significantly attenuated the bilateral hindpaw mechanical allodynia induced by complete Freund’s adjuvant (CFA). The intradiscal CFA injection induced significant decreases in the mechanical withdrawal thresholds of the left (**A**) and right (**B**) hindpaws in response to von Frey filaments (*p* ˂ 0.01), however, no significant bilateral mechanical allodynia were observed in sham-operation groups (the sham exercise and the sham spontaneous recovery) compared to the baseline. For the CFA groups, the bilateral mechanical withdrawal thresholds were significantly increased in the exercise group compared to the spontaneous recovery group on day 28 (*p* ˂ 0.05) and days 42, 56 and 70 (*p* ˂ 0.01). The data are expressed as the mean ± SDs, *n* = 8 in each CFA groups, *n* = 5 in each sham-operation groups. Independent-samples *t* tests were used to examine the differences between the CFA running exercise subgroups *vs.* the CFA spontaneous recovery subgroups, and the sham exercise subgroups *vs.* the sham spontaneous recovery subgroups at the specific time points. We also used one-way analysis of variance (ANOVA) to analyze the within-group differences, and subsequent *post-hoc* tests were used to evaluate the differences between the specific time points and the baseline level in each group. * *p* ˂ 0.05, ** *p* ˂ 0.01 compared to baseline; ^#^ *p* ˂ 0.05, ^##^ *p* ˂ 0.01 CFA exercise group compared to the CFA spontaneous recovery group at the corresponding time points.

Mechanical withdrawal thresholds were determined based on the bilateral hindpaw withdrawal responses to von Frey hair stimulation according to the up-down method described by Dixon [16]. For the CFA groups, the decreased withdrawal thresholds of the bilateral hindpaw peaked at day 3 and were maintained until the end of experiment (day 70 of the CFA spontaneous recovery group). However, no significant changes of withdrawal thresholds were observed in both sham groups (the sham exercise group and the sham spontaneous recovery group) throughout the experiment. These results suggested that the intradiscal CFA injection resulted in mechanical allodynia (*p* ˂ 0.01). Importantly, for the CFA groups, running exercise significantly attenuated the mechanical allodynia from day 28 compared to the spontaneous recovery group (*p* ˂ 0.05) (Figure 1). The left paw withdrawal thresholds of CFA exercise group were significantly increased compared to those of CFA spontaneous recovery group on day 28 (10.6 ± 2.14 *vs.* 5.82 ± 1.72 g, *p* < 0.05), day 42 (14.65 ± 2.19 *vs.* 7.20 ± 1.92 g, *p* < 0.01), day 56 (16.19 ± 1.45 *vs.* 8.14 ± 1.31 g, *p* < 0.01) and day 70 (17.14 ± 1.24 *vs.* 8.60 ± 1.56 g, *p* < 0.01; Figure 1A). The right paw withdrawal thresholds exhibited a pattern similar to those of the left side (Figure 1B). These data suggest that the continuous running exercise helped to attenuate the allodynia induced by CFA intradiscal injection.

### 2.2. Running Exercise Restored the Degenerative Intervertebral Discs and Increased Cell Density

**Figure 2:**
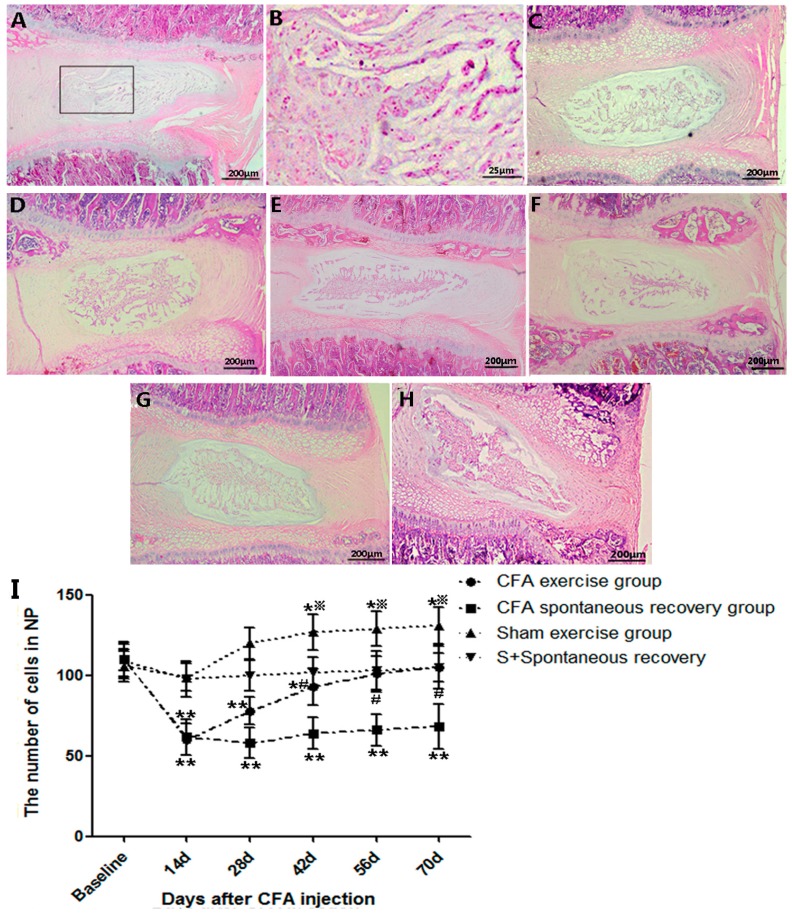

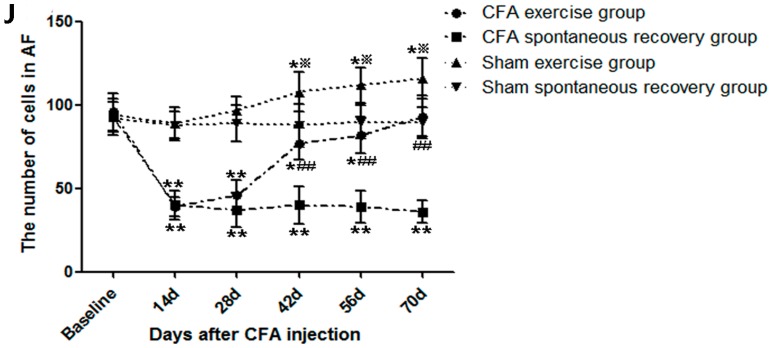
Photomicrographs of midsagittal sections of complete Freund’s adjuvant (CFA)-injected and sham-operation discs. (**A**,**B**) The discs of the CFA model rats exhibited dehydrated nucleus pulposus and blurred boundaries between the nucleus pulposus (NP) and anulus fibrosus (AF) areas at day 14 after CFA injection; (**C**,**D**) Intact NP and the clear distinction between NP and AF in discs of sham exercise groups at day 14 and day 70; (**E**,**F**) The gradual recovery processes were observed at day 42 and day 56 in the CFA exercise group; (**G**) Restored disc structures in the exercise group at day 70 after CFA injection; (**H**) Remarkable degenerative changes in the spontaneous recovery group at day 70 after CFA injection; (**I**,**J**) Quantitative results of NP and AF cell numbers in the CFA groups and sham-operation groups. The CFA exercise group revealed significant increases in both the NP (0.5-fold, *p* ˂ 0.05) and AF (1.4-fold, *p* ˂ 0.01) areas compared to the CFA spontaneous recovery group at day 70. The sham exercise group also showed significant increases of NP and AF compared to the baseline from day 42 (*p* ˂ 0.05) and maintained till the day 70. The sham spontaneous recovery group did not show obvious changes in NP or AF cell numbers throughout the experiment. The data are expressed as the means ± SDs, *n* = 8 in each CFA groups, *n* = 5 in each sham-operation groups. Independent-samples *t* tests were used to examine the differences between the CFA running exercise subgroups *vs.* the CFA spontaneous recovery subgroups, and the sham exercise subgroups *vs.* the sham spontaneous recovery subgroups at the specific time points. One-way analysis of variance (ANOVA) was used to analyze the within-group differences, and subsequent *post-hoc* tests were used to evaluate the differences between the specific time points and the baseline level in each group. * *p* ˂ 0.05, ** *p* ˂ 0.01 compared to baseline; ^#^ *p* ˂ 0.05, ^##^ *p* ˂ 0.01 CFA exercise group compared to the CFA spontaneous recovery group at the corresponding time points. ^※^ *p* ˂ 0.05 sham exercise group compared to the sham spontaneous recovery group at the corresponding time points.

Sections stained with hematoxylin and eosin (HE) were used to evaluate the morphological changes. Sections from rats killed at day 14 after CFA injection exhibited obvious dehydrated nucleus pulposus (NP) and blurred boundaries between NP and anulus fibrosus (AF), which indicated that significant degenerative changes had occurred in CFA groups (Figure 2A,B). The histological examinations of sham exercise groups showed nearly intact structures at day 14 and day 70 (Figure 2C,D). Furthermore, the gradual recovery could be observed in the CFA exercise group as time went by (Figure 2E,F). After running for 8 weeks (day 70), the disc of CFA exercise group revealed a relative normal NP and a distinct boundary between the NP and AF (Figure 2G), while progressions of the decrease in the NP and disorganized lamellae were observed in the CFA spontaneous recovery group at the same time point (Figure 2H). The quantification of the disc cells in the CFA groups revealed a significant decrease in both NP and AF compared to the baseline at day 14 (*p* ˂ 0.01), however, no significant changes were detected in the sham-operation groups at the same time point. Furthermore, the cell counts of the CFA exercise group increased constantly, which showed a 0.5-fold increase of the NP (*p* ˂ 0.05) and a 1.4-fold increase of the AF (*p* ˂ 0.01) compared to the CFA spontaneous recovery group at day 70. In the sham exercise group, the significant increases of NP and AF cells were observed at day 42 (*p* ˂ 0.05) and maintained till the end of experiment, while the numbers of both NP and AF cells remained unchanged in the sham spontaneous recovery group till day 70. (Figure 2I,J).

### 2.3. Running Exercise Promoted Cell Proliferation within the Degenerative Intervertebral Discs

**Figure 3:**
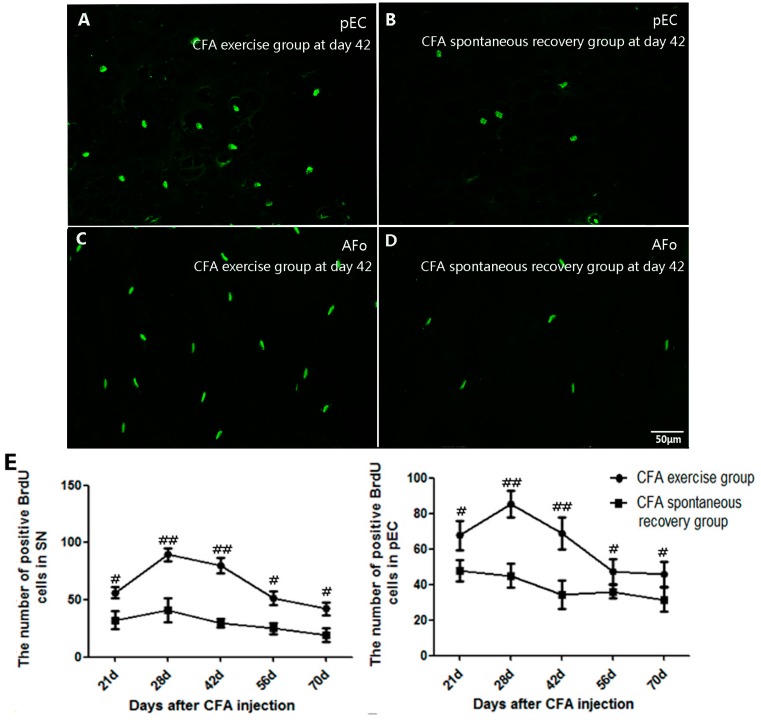

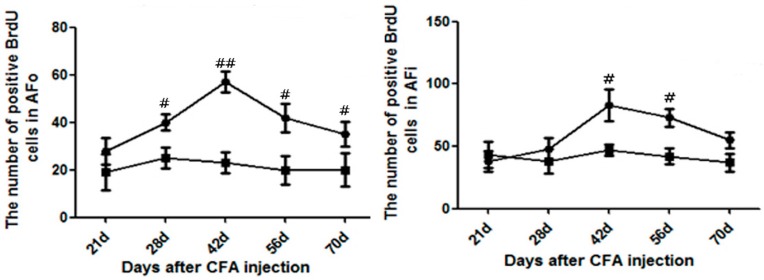
Comparisons of the 5-bromo-2-deoxyuridine (BrdU)-positive cells in four different areas between the CFA exercise group and CFA spontaneous recovery group. (**A**) The peripheral epiphyseal cartilage (pEC) area of the exercise group at day 42; (**B**) The pEC area of the spontaneous recovery group at day 42; (**C**) the outer rings of the AF (AFo) area of the exercise group at day 42; (**D**) the AFo area of the spontaneous recovery group at day 42; and (**E**) Comparisons of the BrdU-positive cells between the CFA exercise group and the spontaneous recovery group. The BrdU-positive cells in the stem cell niche (SN) and pEC of the exercise group were significantly increased from the early time point of day 21 (*p* ˂ 0.05), peaked at day 28 (*p* ˂ 0.01) and were maintained at high levels until the end of the experiment compared to the spontaneous recovery group (*p* ˂ 0.05). The cell numbers in the AFo and the inner rings of the AF (AFi) areas of the exercise group were higher on days 28, 42, 56 and 70 (*p* ˂ 0.05) and peaked at the relatively later time point of day 42 (*p* ˂ 0.01). The data are expressed as the means ± SDs, *n* = 8 in each CFA groups. Independent-samples *t* tests were used to examine the differences between the CFA running exercise subgroups *vs.* the CFA spontaneous recovery subgroups at the specific time points. One-way analysis of variance (ANOVA) was used to analyze the within-group differences, and subsequent *post-hoc* tests were used to evaluate the differences between the specific time points and the baseline level in each group. ^#^ *p* ˂ 0.05, ^##^ *p* ˂ 0.01 compared to the CFA spontaneous recovery group at the corresponding time points.

Proliferating cells with BrdU labeling were detected in four different areas of CFA groups within the IVDs that included the stem cell niche (SN), peripheral epiphyseal cartilage (pEC), outer rings of the AF (AFo) and inner rings of the AF (AFi). The results revealed that the BrdU-positive cells in the pEC area were primarily round in shape and that the proliferation rate was significantly promoted by running exercise. The remarkable increase in the BrdU-positive cells began at day 21 (*p* ˂ 0.05), peaked at day 28 (*p* ˂ 0.01) and was maintained until the end of experiment (*p* ˂ 0.05) compared to the spontaneous recovery group at the corresponding time points (Figure 3A,B,E). Along with the increase in the BrdU-positive cells with elongated or elliptical shapes, we also observed that daily running exercise significantly promoted cell proliferation within the SN, AFo (Figure 3C–E) and AFi areas in similar manners (Figure 3).

### 2.4. Running Exercise Might Attenuate Bilateral Mechanical Allodynia by Increasing Cell Densities

**Figure 4:**
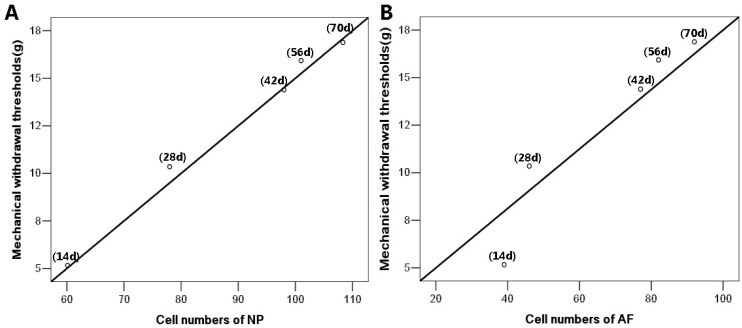
The correlations between the mechanical withdrawal thresholds and the cell counts of CFA exercise group. (**A**) The mechanical withdrawal thresholds were closely related to the increased cell numbers of nucleus pulposus (NP) (*r* = 0.98, *p* ˂ 0.01); (**B**) The tight correlation was also observed in annulus fibrosus (AF) (*r* = 0.96, *p* ˂ 0.01). The correlations between pain relief and cell counts of NP and AF were determined by Spearman rank correlation. *n* = 8 in each CFA exercise group.

To explore the relationship between the pain relief and cell proliferation in the CFA exercise group, we further performed the correlation analysis based on the results of the mechanical withdrawal thresholds and cell counts within NP and AF. The results showed that the withdrawal thresholds of CFA model rats increased positively with the increased cell numbers of both NP (*r* = 0.98, *p* ˂ 0.01) and AF (*r* = 0.96, *p* ˂ 0.01), which indicated that running exercise might attenuate the CFA-induced mechanical allodynia by increasing cell densities within discs (Figure 4).

### 2.5. Discussion

In present study, we used a previously proposed CFA rat model to evaluate the effects of physical exercise on analgesia and degenerative disc restoration. The results revealed that the intradiscal CFA injection induced bilateral hindpaw mechanical allodynia and chronic degeneration within the IVDs, while no degeneration changes were detected in sham-operation groups. Our data demonstrated that daily running exercise significantly relieved the mechanical allodynia and promoted the restoration of the damaged disc structures relative to the controls in CFA groups. Furthermore, the present study provides novel evidence that the reparative effects of physical exercise might have been achieved by stimulating cell proliferation and increasing cell densities in the different areas within degenerative discs. Together, these findings suggest that physical exercise might be an effective approach to the treatment of clinical disc disease.

Chronic pain is an important symptom of DDD, and patients typically present with significantly decreased disc signal intensities on T2-weighted (T2w) magnetic resonance images (MRI) accompanied by reduced disc heights and unclear distinctions between the NP and AF. Additionally, obvious tissue damage induced by degeneration can also be observed upon microscopic examination. Based on previous studies, the CFA model mimics clinical DDD by inducing increases in the release of local inflammatory mediators and degenerative changes that are characterized by decreased NP volumes and distorted AFs within the discs [8,17]. Consistent with the therapeutic effects of physical exercise that have been confirmed in clinical work, the present data also revealed that running exercise significantly ameliorated bilateral allodynia and restored the degenerative discs to normal in a CFA rat model after the eight-week running protocol. Moreover, a previous study found that cell numbers increased by 25% in the AF of normal rats after three weeks of running exercise. Similarly, gradually increased cell numbers were observed in the sham exercise group of the study. In the present study, we also detected markedly increased cell densities of the CFA exercise group; we observed a 0.5-fold increase in the NP and a 1.4-fold increase in the AF compared to the CFA spontaneous recovery at the end of the experiment (day 70). It is known that decreased cell number is generally recognized as one of most dramatic changes in the degeneration process [18], and based on the correlation analysis of the pain relief and cell densities of CFA exercise group, we suggest that the running exercise might have achieved the pain relief and disc restoration by stimulating cell proliferation and increasing cell numbers. Furthermore, we assume that the increased nutritional supply and improved metabolism status that are elicited by physical activity stimulation might have contributed to the cell proliferation and tissue repair, although the exact mechanism is still not fully understood [19,20].

Another important explanation for the increased cell numbers in the CFA exercise group is related to stem cell proliferation and migration. Previous studies have revealed the presence of stem/progenitor cells in particular regions within normal discs of various animals, and the maintenance of stemness implies that these cells have the ability to self-renew to provide specific cell types *in vivo*. Recently, Sasaki *et al.* confirmed that physical exercise positively influences the cell proliferation rate in normal rat IVDs [21]. Since stem cells have also been detected in degenerated human discs, which offers the possibility of achieving disc regeneration via the stimulation of cell proliferation [14,15], we believe that the novel evidences from the degeneration disc model of this study might provide a better understanding of clinical problems. In the present study, the 5-bromo-2-deoxyuridine (BrdU) was used to detect cell proliferation rates, which could be incorporated into the DNA only when the cell undergoes mitosis (at the time of administration). Interestingly, our results showed that the BrdU-positive cell numbers in the SN and pEC areas of the CFA exercise group began to increase on day 21, and these increases were sustained until the end of the experiment, while the cell numbers in the AFo and AFi areas peaked at the later time point of day 42. Several reasons might explain the discrepancies in the proliferation tendencies of the different areas. First, the SN and pEC have been identified as rapid turnover areas in previous studies; thus, the stem cells exhibited relatively rapid proliferation when stimulated by running exercise [21]. Second, the increase in the numbers of positive cells detected at later time points might either have been caused by cell division among slow-cycling cells that were initially marked with BrdU or by cell migration from the SN or pEC areas into this region at a later time point [21]. Although the direct evidence provided in this study is insufficient, our future studies will explore the expressions of progenitor markers and focus on the effects of physical exercise on the division and migration routes of stem cells in degenerated discs.

Although the current study utilized a model that mimicked the clinical course of degenerative disc disease and confirmed that the recovery process benefited from running exercise at the cellular level, it still has several limitations. First, we performed this study in rats, and therefore all data and conclusions should be extrapolated to clinical patients with caution. Second, proliferation rates and disc restoration might vary with the intensity and duration of running exercise; however, this dose-response relationship was not the focus of this study.

## 3. Experimental Section

### 3.1. CFA Injection and Exercise Protocol

The experiments were performed on adult male Sprague Dawley rats weighing 220–250 g. The rats were housed five per cage under a 12-h dark/light cycle with free access to food and water. The experimental procedures were approved by the Animal Ethics Committee of Sun Yat-sen Memorial Hospital and were in accordance with the guidelines for animal usage in research.

The rats were anesthetized intraperitoneally with sodium pentobarbital (30 mg/kg-body weight), and all surgical procedures were performed under sterile conditions. The CFA intervertebral disc degeneration rat model was developed according to the method proposed by Min Lee *et al.* [8]. Via a left transperitoneal approach, the L5-6 IVD was exposed by blunt dissection of the paraspinal muscles, and 10 microliters of CFA (Sigma, St. Louis, MO, USA) were injected with a 26-gauge needle over 10 min at a constant rate. The blood vessels near the spine were avoided with great caution, and the abdominal cavity was irrigated with sterile saline solution in case of any CFA leakage before closing with 3–0 silk sutures. The surgical procedures of sham-operation group were identical to the CFA group, except that CFA was not administered.

**Figure 5:**
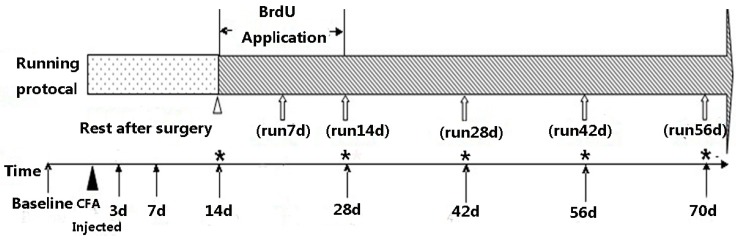
Time course of the experimental protocol ▲ indicates the time point of the complete Freund’s Adjuvant (CFA) injection. △ indicates the starting time of the daily running exercise and the BrdU application. ↑ indicates the time points of the hindpaw withdrawal mechanical threshold measurements. ⇧ indicates the time points of the immunofluorescence staining for the BrdU-positive cells. * indicates the time points of the histological examinations.

Two weeks of rest were allowed after the CFA surgery as a recovery period based on the results of preliminary experiments. At the third week, the 96 CFA rats were divided randomly into the CFA running exercise group (*n* = 48) and the CFA spontaneous recovery group (*n* = 48). *In vivo* labeling with 10 mg/kg/day BrdU (Sigma-Aldrich, Steinheim, Germany) was performed by intraperitoneal injection for 14 days and began at the same time that the running exercise was initiated [22,23]. Another 60 sham-operation rats were divided randomly into sham exercise group (*n* = 30) and sham spontaneous recovery group (*n* = 30). The rats of both CFA exercise group and sham exercise group were trained on two specially designed roller treadmills for 7 days/week, and each treadmill allowed three rats to run simultaneously. Adjustments in the motor speed and exercise duration were implemented via a control panel connected to a genemotor [24,25,26]. To allow for a transition, the daily running duration lasted 20 min/day (9 m/min) for the first week, was increased to 30 min/day (11 m/min) for the second week, and was then increased to 40 min/day (13 m/min) from the third to eighth weeks. The running durations of the subgroups in exercise group varied from 7 days (1 week) to 56 days (8 weeks). Meanwhile, the spontaneous recovery subgroups served as controls at the corresponding time points and were confined to normal cage activity (Figure 5).

### 3.2. Behavioral Studies

The rats were individually placed in a transparent cage with a metal mesh floor and allowed to acclimatize for 20–30 min. An ascending series of von Frey hairs (Stoelting, Wood Dale, IL, USA) were applied perpendicularly to the mid-plantar surface of hind paw, and brisk flinching and paw withdraws were considered positive responses. Each von Frey hair (3.61, 3.84, 4.08, 4.17, 4.31, 4.56, 4.74, 4.93, 5.07, and 5.18) was held for approximately 3–6 s, and 10-min intervals were allowed between each of the stimulation. Testing was performed by an observer who was blind with respect to the group design.

### 3.3. Hematoxylin and Eosin (HE) Staining and Morphological Observation

On days of 14, 28, 42, 56 and 70 after operation, the CFA groups (*n* = 8 for each time point) and sham- operation groups (*n* = 5 for each time point) were killed to observe the morphological changes in the IVDs (Figure 5). The L5-6 IVDs together with the adjacent bilateral half of the vertebral bodies were harvested from the rats and immediately fixed in 10% neutral buffered formalin for 48 h. The IVDs were decalcified with 10% EDTA solution for three weeks and then paraffin-embedded for midsagittal serial sectioning (4.5 μm slice thickness). Sections collected from the IVD samples on days 14, 28, 42, 56 and 70 after the operation were stained with HE using standard protocols [27]. Evaluations of the disc degenerations and cell counts in the AF and NP areas of the CFA group and the sham-operation group were visualized using an Olympus BX63 microscope (Tokyo, Japan), and the pictures were captured with cellSens (Olympus, Tokyo, Japan). Three sections within each IVD sample and three sites each from the NP and AF were randomly selected, and the quantification of the cells was performed at 400× magnification by two blinded observers

### 3.4. Immunofluorescence Staining

Forty rats in the CFA exercise subgroups and 40 rats in the corresponding CFA spontaneous recovery subgroups were sacrificed via overdoses of pentobarbital on days 21 (run 7 days), 28 (run 14 days), 42 (run 28 days), 56 (run 42 days), and 70 (run 56 days) after CFA surgery (Figure 4). The paraffin sections mentioned above were deparaffinized with xylene twice for 10 min and dehydrated with a graded serious of ethanol concentrations of 100%, 95%, 85%, and 75% for 5 min in each solution. Endogenous peroxidase activity was blocked with 0.5% hydrogen peroxide for 10 min, and antigen retrieval was performed by microwave heating in 10 mM citrate buffer (pH 6.0). Next, the sections were incubated in 2 N HCl for 1 h at 37 °C and blocked with 2% BSA/Triton X100 in PBS. The sections were incubated overnight with Anti-BrdU antibody (1:400, Abcam, abcam6326, Cambridge, UK) at 4 °C. The sections were then washed in PBS three times for 10 min each wash and incubated for 1 h at 37 °C with goat anti-rat IgG conjugated with Alex Fluor 488 (1:400, Abcam, ab150157, Cambridge, UK). After counter-staining with DAPI (0.05 μg/mL solution) and cover-slipping, the BrdU-positive cells were visualized with a Nikon Eclipse E600 fluorescence microscope (NIKON, Tokyo, Japan) using NIS-elements software. Sections were treated with the same procedures with the exception that those with the primary antibody excluded served as the negative controls. Three sections within each IVD sample and three sites of four different areas, including the SN, pEC, AFo and AFi, as defined in a previous study, were randomly selected [21]. The BrdU-positive cells were counted at 200× magnification by two blinded observers.

### 3.5. Statistical Analyses

The data were analyzed with SPSS 17.0 (SPSS Inc., Chicago, IL, USA) for Windows, and all measurements are presented as the means ± the standard deviations (SDs). Independent-sample *t* tests were used to examine the differences between the running exercise subgroups and the corresponding spontaneous recovery subgroups at the specific time points. We also used one-way analysis of variance (ANOVA) to analyze the within-group differences, and subsequent post-hoc tests were used to evaluate the differences between the specific time points and the baseline level in each group. The correlations between pain relief and cell counts of NP and AF were determined by Spearman rank correlation. *p* < 0.05 was considered statistically significant.

## 4. Conclusions

In conclusion, running exercise played an important role in reducing allodynia and restoring the discs in a CFA-induced degeneration rat model. After an eight-week running protocol, histological examinations revealed relatively intact structures and significantly higher BrdU-positive cell counts in the exercise group compared to the spontaneous recovery group. These results suggest that running exercise might achieve its analgesic and therapeutic effects at the cellular level, which is promising for the formulation of appropriate therapies for intervertebral disc degeneration.
